# Whole-Genome Sequences of Agricultural, Host-Associated *Campylobacter coli* and *Campylobacter jejuni* Strains

**DOI:** 10.1128/genomeA.00833-16

**Published:** 2016-08-18

**Authors:** Vikrant Dutta, Eric Altermann, Jonathan Olson, Gregory Allan Wray, Robin M. Siletzky, Sophia Kathariou

**Affiliations:** a Department of Food, Bioprocessing and Nutrition Sciences, North Carolina State University, Raleigh, North Carolina, USA; b Rumen Microbiology, AgResearch Grasslands, Palmerston North, New Zealand; c Riddet Institute, Massey University, Palmerston North, New Zealand; d Department of Biological Sciences, North Carolina State University, Raleigh, North Carolina, USA; e Department of Biology, Duke University, Durham, North Carolina, USA

## Abstract

We report here the genome sequences of four agricultural, multidrug-resistant *Campylobacter* spp.: *C. coli* 11601 and *C. jejuni* 11601MD, isolated from turkey cecum and jejunum, respectively, and *C. coli* 6067 and *C. coli* 6461, isolated from turkey-house water and swine feces, respectively. The genomes provide insights on *Campylobacter* antimicrobial resistance and host adaptations.

## GENOME ANNOUNCEMENT

*Campylobacter jejuni* and *C. coli* are leading agents of human foodborne disease that frequently colonize the mammalian and avian intestine (1–3). While certain *Campylobacter* lineages colonize diverse animal hosts, others exhibit preference for certain hosts or environments (3–7). Antimicrobial resistance (AMR) is frequently encountered among *Campylobacter* spp. (8). Much remains to be elucidated about the genomic basis of host preference and AMR in *Campylobacter* spp. of agricultural origin.

We determined draft genomes of four MDR strains from a longitudinal study of *Campylobacter* spp. from conventional turkey and swine farms in eastern North Carolina (9). *C. coli* 11601, resistant to tetracycline (T), streptomycin (S), erythromycin (E), kanamycin (K), nalidixic acid (N) and ciprofloxacin (C), was isolated in 2006 from the cecum of a turkey. On the other hand, *C. jejuni* 11601MD (resistant to T, K, N, C) was isolated from the jejunum of the same turkey. *C. coli* 6067 (resistant to T, N, C) was isolated in 2003 from drinker water in a turkey house and belonged to the turkey-associated "cluster II" lineage (10), while *C. coli* 6461 (resistant to T, S, E) was isolated in 2004 from swine feces and its DNA resisted digestion by MboI, an attribute found frequently in *C. coli* from swine, but not turkeys (11).

Genomic DNA was extracted using the DNeasy blood and tissue kit (QIAGEN, Valencia, CA, USA). DNA library preparation used the GS FLX Titanium rapid library preparation kit (Roche, Basel, Switzerland), and sequencing employed the 454 GS FLX high-throughput DNA sequencer (Roche). The raw reads were *de novo* assembled using Newbler version 1.1.03.24 with >30× estimated coverage, <77 contigs, *N*_50_ > 135,251 bp, providing draft genomes with total sizes of 1,695,750, 1,792,449, 1,957,712, and 1,736,776 bp for *C. coli* (6067, 6461, and 11601) and *C. jejuni* (11601MD) strains, respectively. Annotations were performed using the NCBI Prokaryotic Genome Annotation Pipeline (http://www.ncbi.nlm.nih.gov/genome/annotation_prok) and an updated version of the GAMOLA annotation suite (12). Genome annotation identified 1,618, 1,770, 1,939, 1,695 coding sequences, 76 to 123 pseudogenes, 1 to 4 rRNAs, and 35 to 36 tRNAs in *C. coli* 6067, 6461, and 11601 and *C. jejuni* 11601MD, respectively. In *C. coli* 11601 and *C. jejuni* 11601MD, the tetracycline resistance determinant *tet*(O) was plasmid-borne, with one contig of 11601MD harboring exclusively plasmid-associated sequences (13). As previously reported (14), *tet*(O) was found in the chromosome in *C. coli* 6067 and (in a different chromosomal locus) in *C. coli* 6461. Plasmids were also detected in *C. coli* 6067 and 6461.

Potential host association and AMR attributes of such agricultural strains remain poorly understood, with especially limited information from eastern North Carolina, a major turkey- and swine- producing region in the United States. The genome sequences in this announcement can be used to further elucidate population structure, adaptations, and evolutionary biology of agricultural *C. coli* and *C. jejuni* and inform analyses of *Campylobacter* transmission from agricultural reservoirs to the human food supply.

### Accession number(s).

This whole-genome shotgun project has been deposited in DDBJ/ENA/GenBank under the accession numbers LKCQ00000000, LKCR00000000, LKCS00000000, and LKCT00000000. The versions described in this paper are the first versions.
